# Development and Validation of a Predictive Score for Three-Year Mortality in Acute Ischemic Stroke Patients

**DOI:** 10.3390/medicina60091413

**Published:** 2024-08-29

**Authors:** Ioana Cristina Bârsan, Silvina Iluţ, Nicoleta Tohănean, Raluca Maria Pop, Ştefan Cristian Vesa, Lăcrămioara Perju-Dumbravă

**Affiliations:** 1 Faculty of Medicine, Iuliu Hațieganu University of Medicine and Pharmacy, 400012 Cluj-Napoca, Romania; ioana.barsan@umfcluj.ro; 2 Department of Neurosciences, Faculty of Medicine, Iuliu Hațieganu University of Medicine and Pharmacy, 400012 Cluj-Napoca, Romania; tohanean.nicoleta@umfcluj.ro (N.T.); lperjud@umfcluj.ro (L.P.-D.); 3 Department of Pharmacology, Toxicology and Clinical Pharmacology, Iuliu Haţieganu University of Medicine and Pharmacy, 400337 Cluj-Napoca, Romania; raluca.pop@umfcluj.ro (R.M.P.); stefan.vesa@umfcluj.ro (Ş.C.V.)

**Keywords:** acute ischemic stroke, resistin, mortality, predictive score

## Abstract

*Background and Objectives*: Acute ischemic stroke (AIS) is a leading cause of death and disability with poor long-term outcomes. Creating a predictive score for long-term mortality in AIS might be important for optimizing treatment strategies. The aim of this study is to develop and validate a predictive score for three-year mortality in patients with AIS using several demographic, clinical, laboratory and imaging parameters. *Materials and Methods*: This study included 244 AIS patients admitted to a tertiary center and followed up for three years. The patients’ data included demographics, clinical features, laboratory tests (including resistin and leptin levels) and imaging parameters. The patients were randomly divided into a predictive group (n = 164) and a validation group (n = 80). *Results*: Advanced age, a high NIHSS score, low levels of hemoglobin, elevated resistin levels and the presence of carotid plaques were independently associated with three-year mortality. The predictive model incorporated these variables, and it was validated in a separate cohort. Leptin levels did not significantly predict mortality. *Conclusions*: This study developed and validated a promising predictive score for three-year mortality in patients with AIS. Advanced age, high NIHSS scores, low hemoglobin levels, elevated resistin levels and the presence of carotid plaques were the independent predictors of long-term mortality.

## 1. Introduction

Acute ischemic stroke (AIS) is one of the most important causes of death and a major cause of long-term disability worldwide [1]. Despite progress in stroke management, its long-term prognosis remains uncertain. More than two-thirds of patients with AIS are either dead or functionally dependent after five years [2]. Post-stroke mortality is estimated to be around 40.3% at one year and about 51.65% at five years [3,4]. The identification of outcome predictive biomarkers in patients with AIS is essential for prognostic precision and to optimize treatment strategies. Many risk factors appear to affect the long-term outcomes after stroke. Recent studies have identified several factors that influence life expectancy. Among the most significant are female gender and an age over 85 [5]. Moreover, stroke severity, level of consciousness on admission, atherosclerotic coronary and noncoronary vascular disease, cardiac arrhythmia and diabetes have been identified as risk factors that predict mortality and the recurrence of stroke [5,6].

With advancements in understanding the pathophysiologic mechanisms of AIS, a wide range of serum biomarkers have been discovered in the past ten years. These include over 100 different markers related to inflammation, hormones, hemostasis, oxidative stress and metabolism [7]. Among the various potential biomarkers, resistin and leptin, two adipokines involved in inflammation, metabolism and cardiovascular homeostasis, have emerged as candidates of particular interest in the context of ischemic stroke [8].

Resistin is a hormone initially described as originating from the adipose tissue, hence it being classified as an adipokine, but in humans, it is mainly secreted by peripheral blood mononuclear cells like macrophages and monocytes [9]. Resistin is a key regulator of metabolism. In addition, it has been clearly linked to inflammatory processes, as its expression is upregulated by pro-inflammatory cytokines such as interleukin 1 (IL-1), interleukin 6 (IL-6) and tumour necrosis factor alpha (TNF). In turn, resistin itself can stimulate mononuclear cells to express TNF and IL-6 [10]. High resistin levels have been observed in patients with cardiovascular diseases, including stroke, which suggests its potential role in promoting the pro-inflammatory state associated with acute ischemic stroke [11]. The role of resistin in predicting long-term mortality post-AIS is an aspect of ongoing research, with evidence suggesting that high resistin levels may be associated with an increased risk of adverse outcomes, including mortality [12,13]. However, the exact mechanisms through which resistin influences long-term mortality post-AIS remain unclear, requiring further exploration to establish its prognostic utility.

Leptin, another adipokine predominantly produced by adipocytes, regulates energy balance through its role in metabolism and modulating the appetite. It also influences immune responses by exerting a pro-inflammatory effect, inducing the activation of T-lymphocytes, while promoting the transformation of macrophages into foam cells. As with resistin, increased leptin levels appear to promote atherogenesis. It stimulates the proliferation and migration of vascular smooth muscle cells and induces oxidative stress within endothelial cells. Furthermore, leptin has been linked to decreased fibrinolysis, increased platelet aggregation and the development of arterial calcification [14,15,16]. High leptin levels have been significantly associated with the incidence of ischemic stroke in both men and women, independent of other cardiovascular risk factors, but its prognostic role in the outcomes after stroke remains uncertain, as studies have shown both a protective and harmful role [14,17].

The aim of this study is to develop and validate a predictive score for three-year mortality in patients with AIS using several demographic, clinical, laboratory and imaging parameters.

## 2. Materials and Methods

This prospective, observational, analytical cohort study included 244 patients with AIS consecutively admitted to the two Neurology Departments of Cluj-Napoca Emergency County Clinical Hospital between 1 December 2020 and 15 July 2021 who survived hospitalization and were discharged. The diagnosis of AIS was based on clinical assessment and non-injected cerebral computed tomography (CT). This study was conducted in accordance with the Declaration of Helsinki and was approved by the Ethics Committee of Iuliu Haţieganu University of Medicine and Pharmacy, Cluj-Napoca, Romania (No. 278/11.08.2020).

The inclusion criteria were as follows: adults diagnosed with new AIS confirmed by imaging techniques, with written informed consent signed by the patient or close relatives. The exclusion criteria were as follows: patients who died during hospitalization; with hemorrhagic stroke or hemorrhagic transformation; diagnosed with COVID-19 infection, neoplastic pathology or sepsis; with documented autoimmune diseases or dementia; or with imaging results inconsistent with AIS.

Upon admission, all patients underwent a comprehensive clinical evaluation. This included a detailed medical history, neurological examination using the National Institute of Health Stroke Scale (NIHSS) and a general physical assessment, including body mass index (BMI) calculation and Modified Rankin Scale (mRS) scoring. Demographic information such as age and gender was recorded, along with cardiovascular risk factors (smoking, dyslipidemia, diabetes mellitus, arterial hypertension) and comorbidities (e.g., atrial fibrillation, heart failure, ischemic heart disease, heart valve disease). Laboratory tests for leptin, resistin, hemogram, blood urea nitrogen (BUN), creatinine, aspartate aminotransferase (AST), alanine transaminase (ALT) and C-reactive protein (CRP) were also conducted.

Carotid plaques were identified during ultrasound examination based on criteria including a localized protrusion of the carotid wall thicker than 1.5 mm or than 50% of the adjacent intima–media thickness [18]. Using the TOAST classification (Trial of Org 10172 in Acute Stroke Treatment), etiological classification of acute ischemic stroke included atherothrombotic stroke, cardioembolic stroke, small vessel disease, undetermined etiology and other determined causes [19]. The ischemic lesion volume was calculated using the ellipsoid volume formula V = 4/3 π × (A/2) × (B/2) × (C/2), where A is the greatest diameter in the axial plane, B is the diameter at 90° to A in the axial plane and C is the craniocaudal diameter.

Blood samples were collected the morning after admission in EDTA and biochemistry tubes. Plasma was separated by centrifugation within 30 min and stored at −80 °C until analysis. Resistin and leptin levels were measured using ELISA kits, following the manufacturer’s instructions. All the absorbance readings were carried out using a microplate reader, and mortality was tracked for up to three years after discharge.

The patients were randomly divided into two groups. The first group consisted of 164 patients and was used to calculate the predictive score. The second group consisted of 80 patients and served as the validation group.

Statistical analysis was performed using MedCalc^®^ Statistical Software version 22.021 (MedCalc Software Ltd., Ostend, Belgium; https://www.medcalc.org; 2024). The sample size was calculated from a pilot study (7 deceased and 35 survivors). The mean difference between the two groups for the resistin values was 9.53 ng/mL. For a type 1 (α) error of 0.1 and a type 2 (β) error of 0.05, a sample size of 34 patients in the deceased group and 170 patients in the survivor group was calculated. Nominal data were expressed using frequency and percentages. Continuous data were characterized by medians and the 25th–75th percentiles, as the data were not normally distributed according to the Shapiro–Wilk test. The Mann–Whitney test was used for comparisons between groups of quantitative variables, and chi-square tests were employed for comparisons between groups of qualitative variables. Univariate logistic regression was used to calculate the odds ratio (OR). ROC analysis was used to establish a cutoff value for the association of several quantitative variables with death. Variables that achieved statistical significance in the univariate analysis were included in the multivariate logistic regression. Binary logistic regression analysis was employed to determine which variables were independently associated with mortality. A predictive score was calculated using the results from the logistic regression. Statistical significance was considered at a *p* value of <0.05.

## 3. Results

The comparison between the prediction group and the validation group showed no significant differences between the groups (Table 1).

We recorded 64 (39%) deaths in the predictive group. Table 2 contains comparisons between the survivors and deceased. The deceased patients were older and more often female; had a larger lesion volume, higher NIHSS or mRS scores, higher BUN, AST and resistin values, low hemoglobin values and AF or HF; and were likely to have carotid plaques.

For age, a cutoff value of 75 years was calculated for 3-year mortality (area unde the curve (AUC) = 0.785 (95% CI 0.714–0.845); Se = 71.8 (95% CI 59.2–82.4), Sp = 76 (95%CI 66.4–84.0); *p* < 0.001). For resistin, a cutoff of 11.8 ng/mL was calculated for 3-year mortality (AUC = 0.617 (95% CI 538–0.691); Se = 67.1 (95% CI 54.3–78.4), Sp = 55 (95%CI 44.7–65.0); *p* = 0.008). For hemoglobin, a cutoff of 13.4 (g/dL)_was calculated for 3-year mortality (AUC = 0.640 (95% CI 0.561–0.713); Se = 56.2 (95% CI 43.3–68.6), Sp = 67 (95% CI 56.9–76.1); *p* = 0.001). For AST, a cutoff of 26 U/L was calculated for 3-year mortality (AUC = 0.598 (95% CI 0.518–0.673); Se = 48.4 (95% CI 35.8–61.3), Sp = 70 (95%CI 60.0–78.8); *p* = 0.03). For BUN, a cutoff of 40 mg/dL was calculated for 3-year mortality (AUC = 0.661 (95% CI 0.583–0.733); Se = 62.5 (95% CI 49.5–74.3), Sp = 68 (95% CI 57.9–77.0); *p* < 0.001). For mRS, a cutoff of 1 was calculated for 3-year mortality (AUC = 0. 654 (95% CI 0.576–0.726); Se = 59.3 (95% CI 46.4–71.5), Sp = 66 (95% CI 55.8–75.2); *p* < 0.001). For the NIHSS, a cutoff value of 8 was calculated for 3-year mortality (AUC = 0.787 (95% CI 0.716–0.847); Se = 59.3 (95% CI 46.4–71.5), Sp = 88 (95% CI 80.0–93.6); *p* < 0.001). For lesion volume, a cutoff value of 16.8 was calculated for 3-year mortality (AUC = 0.598 (95% CI 0.518–0.673); Se = 50 (95% CI 37.2–62.8), Sp = 71 (95% CI 61.1–79.6); *p* = 0.03).

Logistic regression was used to find out which variables were independently associated with mortality (Table 3). Advanced age, an NIHSS score higher than 8, lower levels of hemoglobin, higher resistin levels and the presence of carotid plaques were independently associated with 3-year mortality. An mRS score higher than 1 and the presence of HF were associated with mortality, but the statistical significance threshold was slightly passed. Neither gender nor the presence of AF nor higher AST or BUN levels were significantly linked to mortality. The predictive score was calculated based on the results of the logistic model. A cutoff value of 0.992 was calculated for predicting mortality (AUC = 0.888 (95% CI 0.829–0.932); Se = 78.1 (95% CI 66.0–87.5), Sp = 87 (95% CI 78.8–92.9). A second predictive score was calculated without taking into account the resistin values. The nonresistin score had an AUC of 0.870 (95%CI 0.809 to 0.918), a Se of 90 (95%CI 80.7–96.5) and an Sp of 70 (95%CI 60.0–78.8). The differences between the AUCs of the two scores slightly surpassed the statistical significance threshold (*p* = 0.1).

The predictive score was prospectively validated in a group of 80 patients with AIS who were followed up for three years. There were no statistically significant differences between the prediction group and the validation group (Table 3). Using the same cutoff value as in the prediction group, the AUC values were as follows: 0.778 (95% CI 0.671–0.863), Se = 73.33 (95% CI 54.1–87.7), Sp = 70 (95% CI 55.4–82.1). There were no statistically significant differences between the AUC values in the two groups (*p* = 0.06).

## 4. Discussion

The present study aimed to develop and validate a predictive score for three-year mortality in patients with AIS. Several demographic, clinical, laboratory and imaging parameters were used in order to create a complex predictive model. The variables that were independently associated with long-term mortality in AIS patients were advanced age, a high NIHSS score, the presence of carotid plaques, a low hemoglobin value and resistin levels. This study’s results confirm that a predictive model based on commonly utilized clinical variables is effective, with good accuracy, and can be applied even in hospitals/outpatient clinics where advanced biomarker analysis is not feasible. However, our study also indicates that incorporating resistin enhances the model’s predictive power and could be a valuable addition to a predictive score. Although resistin and leptin have been studied in relation to the risk of stroke and mortality in patients with AIS, our study is, to the best of our knowledge, the first to incorporate resistin into a validated predictive score for three-year mortality in AIS patients. The validation of our predictive model might offer clinicians a practical tool for stratifying mortality risk and modifying the follow-up in AIS patients, as an accurate prediction of long-term mortality will help choose the right patients for the implementation of secondary prevention strategies.

There are several predictive scores or nomograms for long-term mortality after AIS [20,21,22]. However, most of the recent studies have several important drawbacks that may not make them a good tool for clinical practice. Many of them have a retrospective design, and some of them have not accounted for several important variables like smoking or imaging parameters and also introduced cancer patients into the study. The complexity of cancer patients (type of cancer, stage, type of treatment) makes them a challenging population to employ in developing a predictive score/nomogram for stroke patients; therefore, we excluded them. The large majority of the predictive scores focus only on very short-term mortality (in-hospital, 1–6 months), but there are few concentrating on medium-term (1 year) or long-term mortality (3–5 years). Axford et al. [22] developed three predictive models for three-year mortality after AIS, with the AUCs ranging from 0.743 to 0.777. In a study by Ramiro et al. [23], the integration of biomarkers such as endostatin, IL-6 and TNF-R1 with clinical variables achieved an AUC of approximately 0.75 for predicting long-term mortality (5 years) after ischemic stroke. A study by Lin et al. [20] aimed to develop a novel score for predicting one-year mortality after AIS and produced a score with a C-index of 0.820 in the development cohort and 0.816 in the validation cohort. Abedi et al. [24] built a predictive model for three-year mortality after stroke with an AUC of 0.77. The predictive score from our study produced an AUC of 0.888, which is higher than those reported in the literature. The study population in many of these studies has predominantly been Asian, which may differ from European populations in terms of their genetics and environment. Almost all studies included patients before the COVID-19 pandemic. This poses serious issues due to the fact that the pandemic overwhelmed healthcare systems, with reduced access to emergency units, leading to delays in diagnosis, treatment and rehabilitation and other challenges that worsened the outcomes of AIS patients [25,26,27]. During the COVID-19 pandemic, heart disease, diabetes, lung conditions and a sedentary lifestyle became more prevalent or more severe than in the pre-pandemic population [28]. Models developed from pre-pandemic data are unlikely to reflect the current situation. The predictive score from our study produced an AUC of 0.888, higher than the ones reported in the literature. Our study included patients from a period during which the COVID-19 pandemic was at its peak. Also, there have been no other validated predictive models (pre- or post-pandemic) that have used highly specific biomarkers, like resistin or leptin, for long-term mortality in AIS patients.

Resistin is one of the components of the predictive score developed and validated in this study. Resistin has been studied primarily in relation to the risk of AIS, but there are few studies examining its association with the long-term outcomes of AIS patients. Bouziana et al. [15] found that resistin levels were associated with a worse long-term functional outcome but not with mortality. In a recent meta-analysis, resistin was associated with a higher risk of stroke but not with mid-term or long-term mortality, although this was the situation for other adipokines [29]. Efstathiou et al. [30] showed that high resistin levels are independently associated with an increased risk of 5-year mortality or disability after AIS. However, there is no predictive score that includes resistin as a variable. The mechanisms by which resistin may influence long-term mortality in AIS patients are multifaceted, involving both inflammatory and cardiovascular pathways [31]. Resistin is a pro-inflammatory adipokine secreted by macrophages, and it plays a significant role in the pathophysiology of atherosclerosis and cardiovascular diseases. Atherosclerosis and cardiovascular disease are closely linked to the short-term and long-term outcomes of AIS. Atherosclerosis is characterized by a high degree of inflammation, which promotes monocytes’ migration to the arterial endothelium, where they transform into macrophages. The macrophages will absorb lipoproteins, transforming into foam cells, which can contribute to plaque formation. Resistin is also produced by macrophages, in addition to adipocytes, and it induces cytokine and chemokine expression [32,33,34]. Resistin contributes to atherosclerosis through inducing the migration of monocytes to the arterial endothelium, where they differentiate into macrophages. The integrated macrophages will absorb oxidized low-density lipoproteins, which will lead to the formation of foam cells and arterial plaque development [35,36]. Resistin further exacerbates inflammation by inducing the expression of pro-inflammatory cytokines and chemokines such as IL-6, TNF-α and Monocyte Chemoattractant Protein-1 (MCP-1) [37]. The exacerbation of inflammation can destabilize atherosclerotic plaques, increasing the risk of plaque rupture, which can precipitate ischemic events. Resistin promotes endothelial dysfunction by increasing the expression of adhesive molecules (intercellular adhesion molecule 1 (ICAM-1) and vascular cell adhesion molecule 1 (VCAM-1))), facilitates the adhesion of monocytes to the endothelium and increases the permeability of endothelial cells, which means that more lipoproteins penetrate the vessel wall and contribute to plaque formation [38,39]. Resistin decreases the production of nitric oxide, an important vasodilator and anti-inflammatory mediator, and in this way, it impairs endothelial function and promotes vasoconstriction and thrombosis [40]. Resistin also seems to be implicated in myocardial remodeling, as some studies show that high resistin level is associated with diastolic dysfunction and delayed left ventricular relaxation and promotes cardiac hypertrophy, which can lead to HF, a disease with high mortality frequently encountered in AIS patients [41,42,43]. By promoting cardiac hypertrophy and remodeling, resistin contributes to reducing cardiac output, which, in turn, exacerbates low cerebral blood flow and increases the likelihood of adverse outcomes post-stroke [44]. The persistent elevation of resistin after an acute stroke may reflect ongoing inflammatory and vascular stress, predicting a higher risk of mortality due to cardiovascular complications. High levels of resistin have been recorded in patients with cardiovascular disease and after major cardiovascular events [21,33,41,43,45,46]. The persistent elevation of resistin after an acute stroke may reflect ongoing inflammatory and vascular stress, predicting a higher risk of mortality due to cardiovascular complications [47]. Resistin may also influence the outcomes in AIS patients through its role in neuroinflammation. Elevated resistin levels have been associated with the increased expression of pro-inflammatory cytokines within the central nervous system, besides a systemic effect, which can contribute to secondary brain injury following AIS. Resistin can facilitate the infiltration of leukocytes into the stroke location, which can further augment local inflammation and damage the blood–brain barrier [48]. This neuroinflammatory response may impair neuroplasticity and recovery, leading to worse functional outcomes and higher long-term mortality [49].

Leptin levels were not statistically significantly associated with mortality in our study, although the deceased group registered lower levels. Leptin was included in this study due to its well-documented but controversial involvement in stroke and its potential role as a biomarker for inflammation and atherosclerosis. The hypothesis was that leptin could contribute to the prognosis of AIS patients. Leptin has pro-inflammatory effects, which can influence the cardiovascular system [50]. It can stimulate the production of reactive oxygen species and promote the overexpression of inflammatory cytokines, such as TNF-α and IL-6 [51]. These effects contribute to the development and progression of atherosclerosis and thus can worsen the long-term outcomes of AIS patients. Leptin affects endothelial cell function, promotes angiogenesis and increases platelet aggregation [52]. The relationship between leptin levels and long-term mortality after AIS is complex and not fully elucidated. Some studies suggest that high or low leptin levels may be linked to an increased risk of severe AIS and a poor prognosis, probably due to its pro-inflammatory effects and its role in atherosclerosis, but others have not found any significant correlation between leptin levels and long-term mortality after a stroke [17,30,53,54,55]. This inconsistency may arise from the hormone’s dual role in inflammation and metabolism, as well as variations in the study designs, population types and testing methods. The effects of leptin might be mediated or masked by the presence of other biomarkers, such as resistin, or comorbidities, like HF, which showed a stronger association with mortality in this study. Future research should explore leptin’s role in specific subgroups of AIS patients or in combination with other biomarkers to understand its prognostic potential better.

In our model, advanced age, a high NIHSS score, the presence of carotid plaques and low hemoglobin levels were strong predictors of three-year mortality after AIS. A high mRS score and the presence of HF were marginally associated with mortality. All of these variables have a combined impact on the body’s ability to sustain a major impact like AIS and make the recuperation process very difficult. A study by Novbakht et al. [56] found that patients aged over 75 years had an almost 4 times higher risk of mortality, which is very similar to the findings of our study. Other studies have shown that age is associated with long-term mortality [23,57]. A high NIHSS score signifies greater neurological impairment, which indicates a more severe brain injury and is directly correlated with poor outcomes and high mortality rates. Several studies have proven that NIHSS score is a powerful predictor of short- and long-term mortality [30,58,59,60]. Studies regarding anemia in AIS patients showed increased mortality in patients with low hemoglobin levels [6,61,62]. The presence of carotid plaques is associated with poor outcomes in AIS patients [63,64]. HF further complicates the recovery and outcomes after AIS, as these two conditions have a bidirectional association. The mortality after AIS was described as being up to 4.5 times higher in patients with HF compared to controls, a number close to our data [65].

This study has several important strengths: the prospective design provided more reliable findings; the inclusion of resistin as a biomarker has added a significant novel contribution to the stroke prognosis research; the patients were recruited exclusively during the COVID-19 pandemic, providing insights into the contemporary situation; and we conducted successful validation of the predictive model in an independent cohort. The limitations of this study include it being a single-center study and the size of the validation cohort, which may limit the generalizability of our findings. This study was conducted during the initial two peaks of the COVID-19 pandemic. The pandemic induced significant challenges for healthcare management, which affected the recruitment of patients, the data collection and the follow-up protocol, reducing our ability to assemble a larger validation cohort.

Future research will focus on conducting a multi-center study to increase the generalizability of the predictive score. By involving multiple centers, the study will be able to include a more diverse patient population and will enhance the applicability of the predictive score. The inclusion of other biomarkers, such as additional adipokines, and the application of more advanced imaging techniques, such as radiomics, could significantly enhance the accuracy of the predictive score. As the multi-center study progresses, we anticipate including a much larger cohort of patients. This larger sample size will enable the integration of the predictive score with machine learning models, increasing the accuracy of the score. Also, there is the possibility of extending the current study with a follow-up period of five years. 

## 5. Conclusions

This study developed and validated a promising predictive score for three-year mortality in patients with AIS. Advanced age, high NIHSS scores, low hemoglobin levels, elevated resistin levels and the presence of carotid plaques were the independent predictors for long-term mortality. Leptin levels were not proven to be a predictor of AIS mortality. Although future research is needed, by incorporating resistin as a biomarker, in addition to the traditional clinical markers, this score represents an important step and could improve the long-term management and prognosis of AIS patients.

## Figures and Tables

**Table 1:** Comparison between predictive group and validation group.

Variables			Validation Group (n = 80)	Score Group (n = 164)	*p*
Deceased	No		50 (62.5%)	100 (61%)	0.92
	Yes		30 (37.5%)	64 (39%)	
Age (years) *			72 (63;79)	71 (62;80)	0.48
Sex		Female	49 (61.3%)	80 (48.8%)	0.09
		Male	31 (38.8%)	84 (51.2%)	
BMI (kg/m^2^) *			27.9 (24.8;31.2)	27.3 (24.6;30.0)	0.52
Smoking		No	50 (62.5%)	90 (54.4%)	0.27
		Yes	30 (37.5%)	74 (45.1%)	
Alcohol consumption		No	74 (92.5%)	147 (89.6%)	0.62
		Yes	6 (7.5%)	17 (10.4%)	
Obesity		No	56 (70%)	127 (77.4%)	0.27
		Yes	24 (30%)	37 (22.6%)	
AH	No		16 (20%)	33 (20.1%)	1
	Yes		64 (80%)	131 (79.9%)	
AF	No		60 (75%)	133 (81.1%)	0.35
	Yes		20 (25%)	31 (18.9)	
HF	No		73 (91.3%)	145 (88.4%)	0.65
	Yes		7 (8.8%)	19 (11.6%)	
History of MI	No		76 (95%)	150 (91.5%)	0.46
	Yes		4 (5%)	14 (8.5%)	
IHD	No		66 (82.5%)	133 (81.1%)	0.92
	Yes		14 (17.5%)	31 (18.9%)	
Valvulopathy	No		67 (83.8%)	143 (87.2%)	0.59
	Yes		13 (16.3%)	21 (12.8%)	
DM	No		61 (76.3%)	119 (72.6%)	0.64
	Yes		19 (23.8%)	45 (27.4%)	
Dyslipidemia	No		22 (27.5%)	36 (22%)	0.42
	Yes		58 (72.5%)	128 (78%)	
NIHSS score *			5 (4;10)	6 (3;10)	0.50
mRS *			1.5 (1;2)	1 (1;2)	<0.001
Systolic blood pressure (mmHg) *			150 (130;166.)	150 (134.5;164)	0.63
Diastolic blood pressure (mmHg) *			80 (72;92.5)	81 (78.5;90)	0.04
Heart rate (b/m) *			78 (71.5;89.5)	78 (68;90)	0.29
Hemoglobin (g/dL) *			13.6 (12.7;14.7)	13.9 (12.5;15.2)	<0.001
Leucocytes (×10^3^/mL) *			7.8 (6.4;10.1)	8.6 (6.6;10.9)	0.08
Thrombocytes (×10^3^/mL) *			214 (176;281)	218 (177;271)	0.24
AST (U/L) *			26 (20;31)	23 (19;31)	0.08
ALT (U/L) *			19 (13;27)	18 (12.5;28.7)	0.25
BUN (mg/dL) *			40 (30;50.2)	39 (29.5;50)	<0.001
Creatinine (mg/dL) *			0.78 (0.66;1.09)	0.85 (0.71;1.04)	0.04
Glycemia (mg/dL) *			109.5 (91;140)	110 (89.5;136)	0.42
Leptin (ng/mL) *			47.1 (16.3;86.5)	48.5 (18.5;92.5)	0.53
Resistin (ng/mL) *			13.4 (8.9;18.5)	12.5 (8.9;23.3)	0.20
CRP (mg/L) *			0.89 (0.38;4.21)	1.04 (0.2;2.6)	0.79
MCA stroke	No		29 (36.3%)	75 (45.7%)	0.20
	Yes		51 (63.7%)	89 (54.3%)	
Lesion volume (mL)			14.9 (6.3;33.8)	12.6 (8.2;26.4)	0.49
Carotid plaque	No		19 (23.8%)	44 (26.8%)	0.71
	Yes		61 (76.3%)	120 (73.2%)	
Thrombolysis	No		62 (77.5%)	138 (84.1%)	0.27
	Yes		18 (22.5%)	26 (15.9%)	

* Median and 25;75 percentiles; n: number of cases; BMI: body mass index; AH: arterial hypertension; AF: atrial fibrillation; HF: heart failure; IHD: ischemic heart disease; MI: myocardial infarction; DM: diabetes mellitus; NIHSS: National Institute of Health Stroke Scale; mRS: Modified Rankin Scale; AST: aspartate aminotransferase; ALT: alanine transaminase; BUN: blood urea nitrogen; CRP: C-reactive protein; MCA: middle cerebral artery.

**Table 2:** Predictive score group: comparison of survivors and deceased patients.

Variables		Survivors (n = 100)	Deceased (n = 64)	OR (95%CI)	*p*
Age (years) *		65 (60;74)	80 (71;85)	1.14 (1.07–1.16)	**<0.001**
Sex	Female	42 (42%)	38 (59.4%)	2.01 (1.06–3.81)	**0.04**
	Male	58 (58%)	26 (40.6%)		
BMI (kg/m^2^) *		27.5 (25.2;30.8)	26.9 (23.8;29.3)	0.92 (0.86–0.99)	0.07
Smoking	No	60 (60%)	30 (46.8%)	1.7 (0.90–3.20)	0.11
	Yes	40 (40%)	34 (53.1%)		
Alcohol consumption	No	87 (87%)	60 (93.8%)	0.44 (0.13–1.43	0.26
	Yes	13 (13%)	4 (6.3%)		
Obesity	No	75 (75%)	52 (81.3%)	0.69 (0.31–1.5)	0.45
	Yes	25 (25%)	12 (18.8%)		
AH	No	23 (23%)	10 (15.6%)	1.61 (0.71–3.66)	0.34
	Yes	77 (77%)	54 (84.4%)		
AF	No	88 (88%)	45 (70.3%)	3.09 (1.38–6.94)	**0.009**
	Yes	12 (12%)	19 (29.7%)		
HF	No	96 (96%)	49 (76.6%)	7.34 (2.31–23.32)	**<0.001**
	Yes	4 (4%)	15 (23.4%)		
History of MI	No	93 (93%)	57 (89.1%)	1.63 (0.54–4.89)	0.55
	Yes	7 (7%)	7 (10.9%)		
IHD	No	84 (84%)	49 (76.6%)	1.6 (0.73–3.53)	0.32
	Yes	16 (16%)	15 (23.4%)		
Valvulopathy	No	91 (91%)	52 (81.3%)	2.33 (0.92–5.9)	0.11
	Yes	9 (9%)	12 (18.8%)		
DM	No	70 (70%)	49 (76.6%)	0.71 (0.34–1.46)	0.46
	Yes	30 (30%)	15 (23.4%)		
Dyslipidemia	No	18 (18%)	18 (28.1%)	0.56 (0.26–1.18)	0.18
	Yes	82 (82%)	46 (71.9%)		
NIHSS score		4 (2;7)	9.5 (6;14)	1.27 (1.17–1.38)	**<0.001**
mRS		1 (0;2)	2 (1;2.75)	1.72 (1.26–2.36)	**0.001**
Systolic blood pressure (mmHg) *		149 (130;164.5)	151 (140;163.7)	1 (0.99–1.02)	**0.36**
Diastolic blood pressure (mmHg) *		83.5 (80;94)	80 (73.5;90)	0.98 (0.96–1.01)	0.21
Heart rate (b/m) *		77.5 (68;88.7)	81 (67.2;93.2)	1.01 (0.99–1.03)	0.31
Hemoglobin (g/dL) *		14.2 (12.9;15.5)	13.3 (11.5;14.8)	0.76 (0.64–0.9)	**0.003**
Leucocytes (×10^3^/mL) *		8 (6.5;10.5)	9.2 (7.2;11)	1.04 (0.92–1.17)	0.15
Thrombocytes (×10^3^/mL) *		236 (181.5;274)	211 (167.2;277)	0.99 (0.99–1)	0.22
AST (U/L) *		23 (18;28.7)	25.5 (20;32.7)	1.01 (0.99–1.04)	**0.05**
ALT (U/L) *		17 (14;26)	19 (11;30)	0.99 (0.98–1.01)	0.71
BUN (mg/dL) *		35 (28.5;44.7)	42 (35;53)	1.03 (1.01–1.05)	**0.001**
Creatinine (mg/dL) *		0.84 (0.72;1.01)	0.88 (0.69;1.12)	1.01 (0.67–1.52)	0.53
Glycemia (mg/dL) *		107 (89.2;138)	113 (92.5;136.7)	0.99 (0.99–1)	0.72
Leptin (ng/mL) *		56.9 (21.1;92.5)	46.1 (14.9;91.5)	0.99 (0.99–1)	0.21
Resistin (ng/mL) *		11.5 (7.7;22.4)	13.7 (10.6;24.2)	1.02 (1–1.05)	**0.01**
CRP (mg/L) *		0.4 (0.2;1.1)	2.3 (1.3;7.2)	1.31 (1.15–1.49)	**<0.001**
MCA stroke	No	47 (47%)	28 (43.8%)	1.14(0.6–2.14)	0.80
	Yes	53 (53%)	36 (56.3%)		
Lesion volume (mL)		12 (6.2;18.2)	18 (9.9;32.6)	1.03 (1–1.05)	**0.03**
Carotid plaque	No	33 (33%)	11 (17.2%)	2.37 (1.09–5.13)	**0.04**
	Yes	67 (67%)	53 (82.8%)		
Thrombolysis	No	83 (83%)	55 (85.9%)	0.79 (0.33–1.92)	0.77
	Yes	17 (17%)	9 (14.1%)		

* Median and 25–75 percentiles; BMI: body mass index; AH: arterial hypertension; AF: atrial fibrillation; HF: heart failure; IHD: ischemic heart disease; MI: myocardial infarction; DM: diabetes mellitus; NIHSS: National Institute of Health Stroke Scale; mRS: Modified Rankin Scale; AST: aspartate aminotransferase; ALT: alanine transaminase; BUN: blood urea nitrogen; CRP: C-reactive protein; MCA: middle cerebral artery

**Table 3:** Multivariate analysis for mortality at three years.

Variables	B	*p*	OR	95% C.I. for OR	
				Min	Max
Age > 75 years	1.419	**0.005**	4.131	1.526	11.180
Gender	0.437	0.40	1.548	0.552	4.341
AF	0.118	0.84	1.125	0.335	3.774
HF	1.336	0.08	3.803	0.827	17.487
mRS > 1	0.898	0.058	2.454	0.971	6.198
NIHSS > 8	2.261	**<0.001**	9.592	3.396	27.096
Hemoglobin < 13.4 mg/dL	0.989	**0.03**	2.689	1.059	6.824
AST > 26 U/L	0.250	0.59	1.283	0.513	3.209
BUN > 40 mg/dL	0.698	0.12	2.009	0.821	4.915
Resistin > 11.8 mg/mL	1.233	**0.01**	3.431	1.331	8.845
Carotid plaque	1.157	**0.04**	3.180	1.019	9.927
Constant	0.300	0.50	1.350		

B—the regression coefficient; *p*—statistical significance. AF: atrial fibrillation; HF: heart failure; mRS: Modified Rankin Scale; NIHSS: National Institute of Health Stroke Scale; AST: aspartate aminotransferase; BUN: blood urea nitrogen

## Data Availability

The datasets presented in this article are not readily available because the data are part of an ongoing study.
